# Next-Generation Sequencing of *Coccidioides immitis* Isolated during Cluster Investigation

**DOI:** 10.3201/eid1702.100620

**Published:** 2011-02

**Authors:** David M. Engelthaler, Tom Chiller, James A. Schupp, Joshua Colvin, Stephen M. Beckstrom-Sternberg, Elizabeth M. Driebe, Tracy Moses, Waibhav Tembe, Shripad Sinari, James S. Beckstrom-Sternberg, Alexis Christoforides, John V. Pearson, John Carpten, Paul Keim, Ashley Peterson, Dawn Terashita, S. Arunmozhi Balajee

**Affiliations:** Author affiliations: Translational Genomics Research Institute, Phoenix, Arizona, USA (D.M. Engelthaler, J.A. Schupp, J. Colvin, S.M. Beckstrom-Sternberg, E.M. Driebe, T. Moses, W. Tembe, S. Sinari, A. Christoforides, J.V. Pearson, J. Carpten, P. Keim);; Centers for Disease Control and Prevention, Atlanta, Georgia, USA (T. Chiller, S.A. Balajee);; Northern Arizona University, Flagstaff, Arizona, USA (S.M. Beckstrom-Sternberg^,^ J.S. Beckstrom-Sternberg, P. Keim);; Los Angeles County Department of Public Health, Los Angeles, California, USA (A. Peterson, D. Terashita)

**Keywords:** Fungi, next generation sequencing, Coccidioides, genotyping, molecular epidemiology, whole genome sequence typing, research

## Abstract

Next-generation sequencing enables use of whole-genome sequence typing (WGST) as a viable and discriminatory tool for genotyping and molecular epidemiologic analysis. We used WGST to confirm the linkage of a cluster of *Coccidioides immitis* isolates from 3 patients who received organ transplants from a single donor who later had positive test results for coccidioidomycosis. Isolates from the 3 patients were nearly genetically identical (a total of 3 single-nucleotide polymorphisms identified among them), thereby demonstrating direct descent of the 3 isolates from an original isolate. We used WGST to demonstrate the genotypic relatedness of *C. immitis* isolates that were also epidemiologically linked. Thus, WGST offers unique benefits to public health for investigation of clusters considered to be linked to a single source.

Genotyping of microorganisms typically relies on comparison of genomic features (e.g., fragment size, repeats, single-nucleotide polymorphisms [SNPs]) between strains and/or against a database of feature profiles (e.g., PulseNET and mlst.net) for a population of the microbe of interest. Such genotyping tools are useful for molecular epidemiologic studies, microbial forensics, and phylogenetic applications. Molecular epidemiology methods may differ in genotyping specificity in linking cases to sources in an epidemiologic investigation; may be less than optimal (e.g., use of pulse-field gel electrophoresis to identify sources of foodborne outbreak often includes nontarget isolates); may not be sensitive enough to detect minor mutations in closely related strains in a forensic investigation (e.g., identifying markers in nearly identical strains of *Bacillus anthracis*); or may not have the resolution necessary to clearly elucidate population structure (e.g., use of nonphylogenetically informative characters such as amplified fragment-length polymorphism fragments or variable-number tandem repeats to establish clades of organisms).

Next-generation sequencing technology (next gen) provides rapid, relatively cost-effective whole-genome sequence typing (WGST). Although these technologies are relatively novel, they are quickly being adapted for use in the fields of genomics, transcriptomics, and phylogenetics and have been highly successful for resequencing, gene expression, and genomic profiling projects (*1*). Recently, next gen sequencing has been described as a viable genotyping tool in the fields of infectious disease epidemiology and microbial forensics (*2**,**3*).

Coccidioidomycosis is an invasive fungal infection caused by the dimorphic fungus *Coccidioides* spp. and is endemic to the southwestern United States (*4*). Organ donor–transmitted coccidiodomycosis was first reported almost 5 decades ago and is a rare but serious complication of solid organ transplantation; death rate associated with disseminated disease in this patient population is high (72%) (*5*). In these cases, donor-transmitted coccidioidomycosis was recognized because recipients underwent transplantation in a coccidioidomycosis-nonendemic area and had no prior travel history to a coccidioidomycosis-endemic area. No genotyping methods were used to confirm the genetic relationship between isolates recovered from the donor and recipient in any of these studies.

We describe the use of WGST to genotypically link *C. immitis* isolates recovered from a transplant-related cluster of coccidioidomycosis in an area to which it is endemic. Results show that isolates recovered from the transplantation patients were essentially genetically indistinguishable, thereby identifying the donor as the common source for these infections.

## Methods

### Patients and Isolates

In early 2009, coccidioidomycosis was diagnosed for 3 patients (X, Y, and Z); all had recently received transplanted organs in Los Angeles, California, USA, where this fungus is endemic. Later serologic investigations showed that the donor’s postmortem serum was positive for immunoglobulin M antibodies to *Coccidioides* spp.; however, no isolate was available from the donor. Isolates B7709, B7556, and B7557 were available from patients X, Y and Z, respectively, for further molecular analyses.

### Whole-Genome Sequencing

Genomic DNA extracted from the 3 isolates was plated onto potato dextrose agar plates for 5 days for a sterility check. DNA fragment libraries for each of the cluster-associated *C. immitis* strains were constructed for sequence analysis on the SOLiD sequencing platform (Life Technologies, Foster City, CA, USA) according to the manufacturer’s instructions. Libraries were prepared in equimolar ratios, and sequencing was conducted to 50 bp by using SOLiD V3 chemistry as described (*2*).

### WGST Analysis

The whole-genome sequence (WGS) data for each isolate was aligned to the most recent version of the *C. immitis* RS3 strain sequence (AAEC02000000) (*6*) by using the software program BFAST (*7*) with the following exclusion criteria: 1) indel-containing reads; 2) reads aligning to multiple locations; and 3) reads with mapping and alignment scores <20 and <100, respectively. Because *C. immitis* has a high level of repetitive DNA (17% of genome) (*8*) that could confound SNP analysis, reads that matched >1 location on the RS3 genome were identified and removed before SNP analysis.

The alignment files were then used to identify putative SNPs among the 3 outbreak isolates. An SNP caller application (*9*) was used to identify putative SNPs. To be called an SNP, the position had to have a minimum of 5× coverage. After eliminating any bases with a quality score <20 (as reported by SOLiD) or a mapping score <40 (calculated by BFAST), 90% of the reads had to agree. Identified SNPs were then visually evaluated by viewing the WGS alignment in SolScape, a short-read sequence-alignment viewer developed in house (J. Pearson et al., unpub. tool available on request). Any SNPs identified between the 3 cluster isolates were confirmed by Sanger sequencing by using standard methods.

An additional in-house analysis tool, In Silico Genotyper (S. Beckstrom-Sternberg et al., unpub. data; tool available upon request) was used to identify SNPs between the cluster isolates and 10 additional publically available *C. immitis* WGS datasets: CimmH538, CimmRm2394, CimmRm3703, CimmRS3 (*6*); and RMSCC-3505, -3693, -2395, -3474, -3705, -3377 (*10*). SNP calls were required to have a minimum of 5× coverage, at least 1 read on each strand, have 95% of reads contain the alternate base, and have a SNP quality score of >20, as calculated by SAMtools (*11*). These SNPs were then used for phylogenetic analysis of the 13 combined *C. immitis* sequences. Only SNP loci common to all taxa were included in the analysis. In an attempt to remove SNP loci that might be more subject to genome rearrangements, horizontal gene transfer, and potential repeat induced point mutation processes (*8*), SNP loci falling within genomic regions repeated within the reference genome (RS3) were also excluded from the phylogenetic analysis. Repeat regions were identified by using a pairwise self-comparison of the reference genome (RS3) in MUMmer version 3.22 (*12*). Phylogenetic trees were generated by the maximum-parsimony algorithm in MEGA4 (*13*) with bootstrapping of 1,000 replicates. Loci with missing data were removed before analysis.

## Results

### Whole-Genome Sequencing

**Figure 1:**
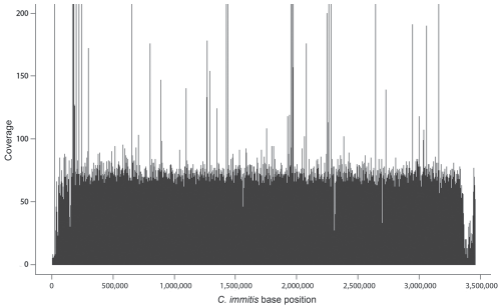
Example coverage plot of sequenced genome of *Coccidioides immitis*. Plot shows base coverage (y-axis) of supercontig 6 from isolate from patient Z, who had coccidioidomycosis. Average depth of coverage for this supercontig was 48.63× over 3,385,806 bases (x-axis) for a total of 164,650,400 bases sequenced.

The generated sequence data (50-bp reads) alignment of the 3 outbreak isolates resulted in average coverage depths of 40.8×, 48.6×, and 33.6× for isolates B7709 (patient X), B7556 (patient Y), and B7557 (patient Z), respectively (Figure 1). The overall percentage of the *C. immitis* RS (revision 3) genome (≈28.9 Mb) coverage by the 3 datasets was similar, ranging from 94.6% to 95.0%. Supercontig 3 had the lowest total base coverage (89.8%–90.3%); supercontig 6 had the highest (96.6%–96.8%).

### SNP Analysis

**Figure 2:**
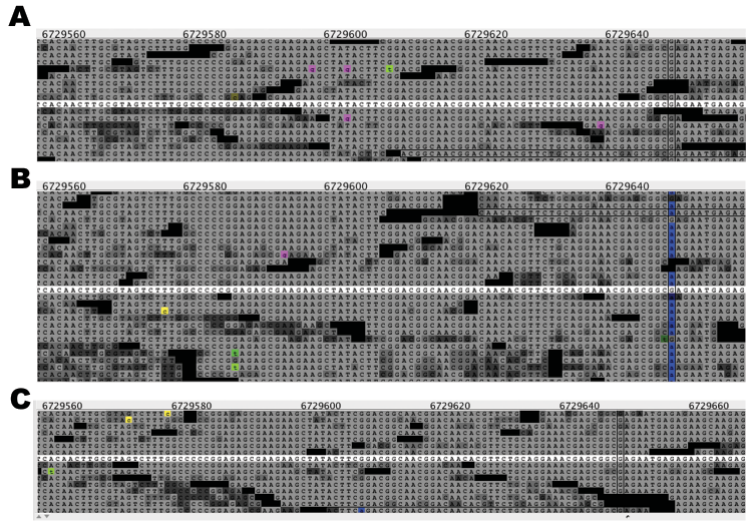
Alignment of *Coccidioides immitis* whole-genome sequence reads flanking a confirmed single-nucleotide polymorphism (RSv3 supercontig 1, position 6729646, highlighted in blue in panel B) among the 3 cluster isolates. Isolates from patients X, Y, and Z, who had coccidioidomycosis, are shown in panels A, B, and C, respectively. The alignment was created by using SolScape, a short-read sequence-alignment viewer developed in house (J. Pearson et al., unpub. data; tool available upon request). Reference sequence position is given at the top of each panel; actual reference sequence is highlighted in white at the center of each panel. Bases differing from the reference sequence are highlighted in pink, green, or yellow.

The initial SNP analysis identified 17 candidate SNPs among the 3 transplant isolates. After further evaluation, including manual inspection of alignments and coverage and, finally, Sanger sequence confirmation, only 3 loci were determined to be polymorphic (see Figure 2 for sequences alignment showing 1 of these SNPs). The isolate from patient X contained 1 SNP, and the isolate from patient Y contained the other 2 SNPs. Comparative SNP analysis of the 13 *C. immitis* genomes showed 32,695 shared SNPs among all taxa. Approximately half (17,080) of these were parsimony informative in that multiple taxa contained alternate allele states; the remaining SNPs (15,615) were considered autapomorphic in that only 1 strain showed the alternate allele state. Of the 32,695 shared SNPs, the cluster isolates differed from the reference genome by an average of 8,541 SNPs.

### Phylogenetic Analysis

**Figure 3:**
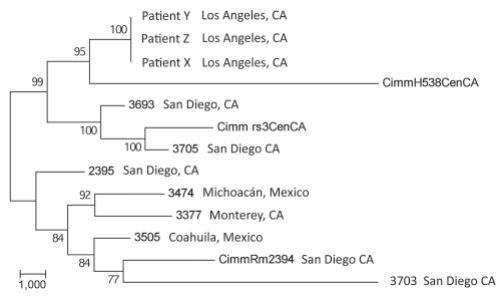
Maximum-parsimony phylogenetic analysis of 13 *Coccidioides immitis* genomes. MEGA4 (*13*) was used to conduct maximum-parsimony analysis of all single-nucleotide polymorphism (SNP) loci common to the 3 transplant isolate genomes and the 10 publicly available *C. immitis* genome sequences (*6*,*10*). A total of 32,695 SNP positions were identified in the final dataset, of which 17,080 were parsimony informative. The percentages of replicate trees in which the associated taxa clustered in the bootstrap test (1,000 replicates) are shown next to the branches. The tree is drawn to scale; branch lengths were calculated by using the average pathway method (*14*) and are in the units of the number of changes over the whole sequence. The consistency index of the tree is 0.63. Scale bar indicates nucleotide substitutions per site.

Maximum-parsimony analysis that used all SNPs common to all 13 taxa is shown in Figure 3. The consistency index (0.63) for the tree indicates a moderate level of homoplasy among these SNPs. However, the high bootstrap values indicate strong support for the outbreak isolates and the central and southern California isolate branch points. Branch lengths indicate that the outbreak isolates are more closely related to the isolates from central California than to the isolates from southern California.

## Discussion

Multiple donor transplant–related coccidioidomycosis cases have been reported (*5**,**15*). In most of these studies, none of the recipients were from *C. immitis*–endemic areas, and the organ donor had either lived in or visited a *C. immitis*–endemic area. For organ transplant recipients living in such areas, coccidioidomycosis is most often believed to occur by primary infection with *Coccidioides* spp. after environmental exposure or from reactivation of latent infection. However, whether additional cases of donor-derived infections are occurring in endemic areas is not clear because the cases are difficult to recognize as such. Molecular epidemiologic tools may help differentiate donor-derived infections from primary or latent infections.

In our investigation, the recipients and the donor were from a *C. immitis*–endemic region, and we used next gen sequencing to conduct WGST to better elucidate the relationship between the isolates recovered in the investigation. Our analyses demonstrate that the *C. immitis* isolates from 3 transplant recipients originated from the same source, the organ donor. Although a molecular clock has not been established for *Coccidioides* spp., we can infer that the minor SNP differences resulted from limited mutation since divergence. Estimated mutation rates in these eukaryotic microbes (≈10^–9^ per base per year) (*16*) limit the possibility of these isolates being direct descendants in clonal lineages.

Previously, only microsatellite-based methods have proven useful for molecular epidemiologic studies of *Coccidioides* spp., which provide adequate separation across geographically diverse samples (*17*) and identifying clonal isolates (genotypically identical) recovered from the same patient (*18*). However, microsatellite methods can be biased in that they may fail to detect genomic changes outside these loci. By using WGST, we firmly established genetic linkage between isolates recovered from patients X, Y and Z, with a total of only 3 SNP differences among the 3 isolates. By comparison, when other *C. immitis* genomes are included in the WGST analysis, we noted 8,700–32,700 SNP differences (Figure 3). We can argue that the recipients may have been infected independent of their receipt of organ transplant, and subsequently, disseminated coccidioidomycosis developed after transplant-associated immunosuppressive therapy. This explanation is plausible given that all 3 recipients lived in an area endemic for *C. immitis*, although less probable given that all 3 received organs from the same donor. However, WGST analyses established that the 3 isolates shared a common ancestry, thereby unequivocally establishing that the isolates originated from 1 donor.

SNPs are highly informative for phylogenetic and epidemiologic analyses. WGST focuses on the SNP differences between all sequenced strains. Although 1 canonical SNP may be all that is required to identify a clonal species, subpopulation, and/or isolate (*19*), the massive number of potential SNPs in a genome provides incredible resolution of nonclonal species as well. By exploring all shared SNPs between a particular group of isolates (e.g., across a species), we are able to not only identify identical or closely related isolates, but also to better understand the population structure for further analyses (e.g., phylogeography) (*20*). As with other genotyping techniques, genotyping fungi (and other eukaryotes) by using SNPs is challenging because of genetic recombination rather than the genetic stability of more clonal microorganisms (i.e., bacteria and viruses) (*21*). Although *Coccidioides* spp. have asexual reproduction, allowing for some clonality, it has extensive recombination, probably from cryptic sexual reproduction (*8**,**22*). The effects of recombination on phylogenetic analyses of *Coccidioides* spp. and similar microbes can be overcome by use of large SNP datasets and appropriate algorithms (*21*). The use of WGST, therefore, provides the highest degree of phylogenetic and genotyping robustness by enabling interrogation of all possible informative SNPs along with other genetic variation (e.g., insertions, deletions, gene changes). The focus of this WGST investigation was limited to SNP analysis, primarily because of sequence coverage of the chosen sequencing method, similar to what has been described as the dirty genome approach (*23*).

Use of WGS for molecular epidemiology has been limited to a handful of studies involving primarily viral pathogens, including linkage of hepatitis C virus strains in humans and wild boars (*24*); genotyping of HIV strains by using near full-length genomes (*25*); and molecular epidemiology of influenza A (H5N1) virus in waterfowl outbreaks (*26*). A more recent study used next gen sequencing to link hospital-associated isolates of methicillin-resistant *Staphylococcus aureus* in Thailand (*3*). We have used WGST to help confirm that the cluster reported here represented donor-transmitted infection and not a primary or latent infection in the transplant recipients. With the wide-scale use of next gen technology for microbe sequencing, we anticipate that WGST will be used more frequently for future public health and forensic applications. The costs per sample are rapidly declining (because of ability to index multiple samples in a single lane [*27*]) and the amount of sequence data per run is greatly increasing (because of improved chemistry) on existing next gen platforms. Third-generation sequencing promises faster turnaround times and exponentially greater read lengths and sequence coverage. These advances will enable sequencing of entire global repositories of pathogens for future WGST analysis. The major challenges to universal acceptance and use of WGST for infectious disease epidemiology are the costs of instrumentation and the development and availability of appropriate bioinformatic tools for data analysis, along with available server/computing capacity. Although the former will depend on the marketplace, the latter is already being addressed by development of novel analysis tools (*7**,**9**,**11**,**28*), global databases (*10*), and access to shared server systems and parallel computing networks (*29**,**30*). These findings also lead us to envision a use for WGS in clinical medicine much sooner than originally anticipated, perhaps within the next 5 years.
